# Nerol Attenuates Ouabain-Induced Arrhythmias

**DOI:** 10.1155/2019/5935921

**Published:** 2019-03-07

**Authors:** José Evaldo Rodrigues de Menezes-Filho, Diego Santos de Souza, Artur Santos-Miranda, Valeska Moraes Cabral, José Nilson Andrade Santos, Jader dos Santos Cruz, Andreza Melo de Araujo, Carla Maria Lins de Vasconcelos

**Affiliations:** ^1^Laboratory of Heart Biophysics, Department of Physiology, Federal University of Sergipe, São Cristóvão, Brazil; ^2^Excitable Membranes Laboratory, Department of Biochemistry and Immunology, Federal University of Minas Gerais, Belo Horizonte, Brazil

## Abstract

Nerol (C_10_H_18_O) is a monoterpene found in many essential oils, such as lemon balm and hop. In this study, we explored the contractile and electrophysiological properties of nerol and demonstrated its antiarrhythmic effects in guinea pig heart preparation. Nerol effects were evaluated on atrial and ventricular tissue contractility, electrocardiogram (ECG), voltage-dependent L-type Ca^2+^ current (I_Ca,L_), and ouabain-triggered arrhythmias. Overall our results revealed that by increasing concentrations of nerol (from 0.001 to 30 mM) there was a significant decrease in left atrium contractile force. This effect was completely and rapidly reversible after washing out (~ 2 min). Nerol (at 3 mM concentration) decreased the left atrium positive inotropic response evoked by adding up CaCl_2_ in the extracellular medium. Interestingly, when using a lower concentration of nerol (30 *μ*M), it was not possible to clearly observe any significant ECG signal alterations but a small reduction of ventricular contractility was observed. In addition, 300 *μ*M nerol promoted a significant decrease on the cardiac rate and contractility. Important to note is the fact that in isolated cardiomyocytes, peak I_Ca,L_ was reduced by 58.9 ± 6.31% after perfusing 300 *μ*M nerol (n=7, p<0.05). Nerol, at 30 and 300 *μ*M, delayed the time of onset of ouabain-triggered arrhythmias and provoked a decrease in the diastolic tension induced by the presence of ouabain (50 *μ*M). Furthermore, nerol preincubation significantly attenuated arrhythmia severity index without changes in the positive inotropism elicited by ouabain exposure. Taken all together, we may be able to conclude that nerol primarily by reducing Ca^2+^ influx through L-type Ca^2+^ channel blockade lessened the severity of ouabain-triggered arrhythmias in mammalian heart.

## 1. Introduction

Cardiac arrhythmia is caused by changes in the coordinated electrical activity of the heart and is surely among the leading causes of sudden death in the modern world [1, 2]. Arrhythmias are generally divided into two major types: (1) abnormalities in impulse generation and (2) conduction disturbances [3]. Reductions in repolarizing outward K^+^ currents and/or augments in depolarizing inward Na^+^ or Ca^2+^ currents can lead to distinct types of cardiac arrhythmias. Cardiac myocytes exhibit an exquisite dynamic control of intracellular Ca^2+^ homeostasis. Perturbations in this control process have been recognized as a major contributor to life-threatening ventricular arrhythmias [4]. In this very aspect the model of arrhythmias induced by exposure of heart tissue to ouabain (which is* per se* a natural product) is an excellent and straightforward approach for the study of the putative antiarrhythmic activity of natural products. Mechanistically, ouabain-triggered arrhythmias is based on disturbances in the intracellular Ca^2+^ handling, overload of Ca^2+^, and unbalance of Na^+^/Ca^2+^ exchanger activity (NCX) [5]. Recent studies have reported that cardiac hypertrophy and heart failure may be associated with increased arrhythmogenic risk by the enhanced NCX activity [6, 7].

Terpenes complain about a large variety of plant-derived substances. Several terpene compounds in the essential oils are monoterpenes and sesquiterpenes [8]. Chemically, monoterpenes are generally characterized by having 10 carbon atoms linked together and two isoprene units [9].

Nerol (C_10_H_18_O) is a monoterpene found in many essential oils [10]. It was originally isolated from the oil of neroli, an oil similar in scent to bergamot oil, which produced orange blossom bergamot (*Citrus aurantium* var. Loved or Bergamia) and is widely used in the production of perfumes [10]. Nerol is the* cis*-isomer of geraniol [11].

Neroli essential oil, containing nerol in its composition, is considered one of the most important aromatics for aromatherapy treatment of heart palpitations, anxiety, and depression resulting from stress and anxiety [12]. It is also effective in diminishing the amplitude of heart muscle contraction, thus benefiting people who suffer from palpitations or coronary artery spasm [13, 14]. Evidence pointed out that neroli essential oil is used in aromatherapy to treat heart palpitations, therefore suggesting its putative effect on the mechanical and/or electrical features of the mammalian myocardium. However, the precise mechanism of action and which compound would be the main responsible remained elusive. The aim of the current study was to investigate the possible effects of nerol on electrical and mechanical activities, and whether it exerts protective effects on ouabain-triggered cardiac arrhythmias in an* ex vivo *model. We, then, hypothesized that nerol could prevent cardiac arrhythmias by modulating Ca^2+^ current and stabilizing intracellular Ca^2+^ homeostasis. In order to test this hypothesis, nerol was administered in isolated mammalian heart preparations and cardiomyocytes.

## 2. Materials and Methods

### 2.1. Animals

To evaluate the effects of nerol on cardiac contractility and on electrocardiogram waveform of intact mammalian heart, experiments were designed and performed using male guinea pigs (*Cavia porcellus*). This investigation was approved by the local Animal Research Ethics Committee of the Federal University of Sergipe, Brazil (Protocol No. 28/12).

### 2.2. Measurement of Contractility in Guinea Pig Left Atrium

Guinea pigs were sacrificed by decapitation and hearts were removed and isolated left atrium mounted vertically in an organ baths containing Tyrode's solution of the following composition (mM): NaCl 120, KCl 2.7, MgCl_2_ 0.9, NaHCO_3_ 11.9, CaCl_2_ 1.37, glucose 5.5, NaH_2_PO_4_ 0.4, pH 7.4. The atria preparations were subsequently connected to Grass FI'-03 (Grass Instruments, USA) force displacement transducers to record changes in atrial contractile force. Each muscle was stretched to the length at which contractile force was maximal (1.0 gf). The atria were electrically paced at 1 Hz with pulses of 1.5 ms duration and stimulated by 70 V pulses. All preparations were allowed to equilibrate for 30 min until complete mechanical stabilization had been achieved. Nerol (≥97%, Lot#MKBP7755V, Sigma-aldrich) was freshly solubilized in 0.5% DMSO and cumulatively added to bath chambers. After each observation, muscles were washed several times and allowed to recover for 30 minutes until their mechanical function completely returned to control values. DMSO at this concentration did not show any significant effect on the variables measured (data not shown, n = 5).

### 2.3. Effect of Nerol on Ca^2+^ Influx

A pharmacological approach was used to investigate the effect of nerol on Ca^2+^ influx. Indeed, concentration-response curves for extracellular CaCl_2_ (0.5 – 8 mM) were obtained before and after preincubating with nerol (300 *μ*M) during 15 min. The results were expressed as percentage of the maximum atrial contractile attained response to a given extracellular CaCl_2_ concentration.

### 2.4. Electrocardiogram Waveform and Left Ventricular Pressure Measurements

Guinea pigs were heparinized (1,000 IU, i.p.) and the hearts excised via an anterolateral thoracotomy and immediately immersed in Tyrode's solution. The aorta was quickly cannulated to the Langendorff apparatus, and the heart was retrogradely perfused (8 mL/min) using a peristaltic pump (Milan Peristaltic Pump, Curitiba, Brazil). Tyrode's solution was maintained at 34.0 ± 1°C (HAAKE F3, Berlin, Germany) and gassed with a mixture of 95% O_2_ and 5% CO_2_ (pH ~ 7.4). To measure the Left Ventricular Developed Pressure (LVDP), a water filled latex balloon was placed into the left ventricular cavity and connected to a pressure transducer (FE221, Bridge Amp, ADInstruments, Colorado, USA). The system was calibrated using a column of mercury 15 cmHg. ECG waveform was recorded through three electrodes (Ag/AgCl/NaCl 1 M) that were placed near the heart surface to detect and record macroscopic electrical signals. All signals were amplified and digitalized sampling frequency of 400 Hz (PowerLab 4/35, ADInstruments, Colorado, USA) and stored in a computer (PC-compatible) for offline processing using dedicated software (LabChart 8 Pro, ADInstruments, Colorado, USA). To measure ECG parameters, such as the PR interval, QT interval, and the duration of the QRS complex, the heart was electrically stimulated by Grass stimulus isolation unit (SIU5) connected to a Grass S48 stimulator (100 V, 1 Hz, 2 ms pulse duration). For QT correction, Bazett's formula was used (QTc = QT/√RR). To measure heart rate (beats per minute), the isolated heart was allowed to beat spontaneously following sinus rhythm.

### 2.5. Effects of Nerol on the L-Type Ca^2+^ Channel in Isolated Ventricular Cardiomyocytes

Using an EPC-9.2 patch-clamp amplifier (HEKA Electronics, Rheinland-Pfalz, Germany) we recorded voltage-dependent L-type Ca^2+^ current (I_Ca,L_) in the whole-cell voltage-clamp configuration of the patch-clamp technique [15, 16]. Ventricular cardiomyocytes were isolated from guinea pig heart by enzymatic digestion as described by Shioya [17] with minor modifications (reduction of enzyme time in the first and second tubes for 6 min). The recording electrodes had uncompensated tip resistances of 1-3 MΩ. Cardiomyocytes presenting a series resistance (Rs) above 10 MΩ were promptly discarded from the analysis. The composition of pipette solution was as follows (in mM): 120 CsCl, 20 TEACl, 5 NaCl, 10 HEPES and 10 EGTA, and 1 MgCl_2_ (pH was set to 7.2 using CsOH) and external solution was as follows (in mM): 150 TEACl, 0.5 MgCl_2_, 1.8 CaCl_2_, 10 HEPES, and 11 glucose (pH 7.4 set using TEA(OH)). The holding potential was set at -80 mV and prepulses were elicited from -80 mV to -40 mV for 50 ms (every 10 s) to inactivate any remnant Na^+^ and/or T-type Ca^2+^ channels [16]. Then, it was applied a test pulse to 0 mV during 300 ms to directly measure I_Ca,L_.

### 2.6. Effects of Nerol on the Ouabain-Triggered Arrhythmias

Antiarrhythmic effect of nerol on the cardiac arrhythmias induced by ouabain was evaluated in spontaneously beating isolated heart [18, 19]. After stabilization (30 min), the hearts were perfused with ouabain (50 *μ*M, [20]) for 10 min. The hearts were preincubated with nerol (either 30 or 300 *μ*M) 10 min before ouabain or nifedipine (0.35 *μ*M). Parameters investigated were maximal inotropic effect, maximal tonotropic effect, inotropic response rate (dP/dt), and onset time of arrhythmias evoked by ouabain. Furthermore, we investigated the occurrence of subtypes of ventricular arrhythmias, such as ventricular premature beats (VPB), ventricular tachycardia (VT), and/or ventricular fibrillation (VF). Arrhythmia scores were evaluated by subtypes and arbitrarily classified as previously validated by Curtis and Walker [21]. Then, The experiment was then divided into 5 intervals of 2 minutes and, depending on the type of arrhythmia, was assigned a scrore. Episodes of VPB < 10 events/2 min were classified as score 0 and > 10 events/2 min scored 1; episodes of VT < 30 s were 2 and > 30 s were 3; episodes of VF < 30 s scored as 4 and > 30 s as 5.

### 2.7. Statistical Analysis

Results were expressed as mean ± SEM. Statistical significance was determined by using Student's *t*-test or one-way ANOVA followed by Tukey's* post hoc* test and Chi-squared test. p < 0.05 was considered significant.

## 3. Results

**Figure 1:**
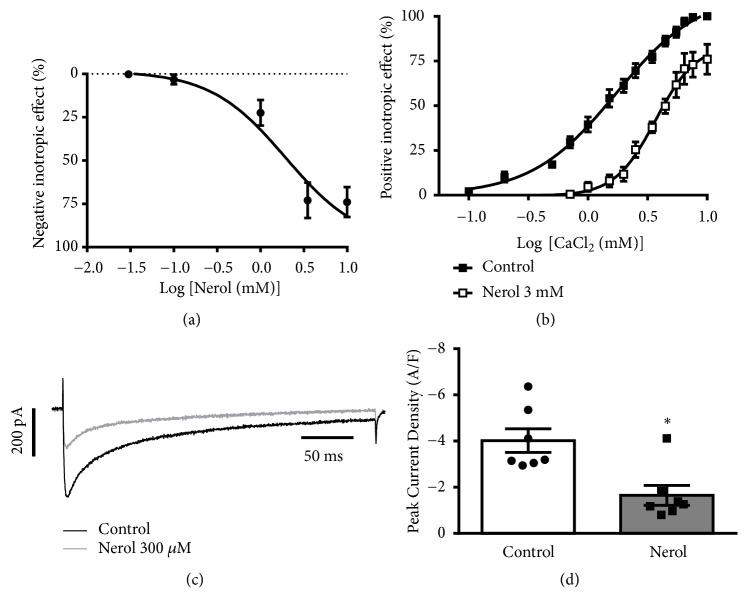
*Effects of nerol on the contractility of guinea pig left atrium and on the calcium influx. *(a) Concentration-response curve for the negative inotropic effect of nerol (EC_50_ = 1.94 ± 0.2 mM, n = 5). (b) Concentration-response curves of CaCl_2_ in control situation and in the presence of nerol (n = 5). (c) Representative recording showing the L-type calcium currents (I_Ca,L_) in control and 300 *μ*M nerol. (d) Average effect of nerol on the I_Ca,L_ density (n = 7, *∗*p<0.05). Data are represented as mean ± SEM. Paired Student's* t*-test.

Our initial series of experiments were aimed at determining the baseline effects of nerol on cardiac contractility. As shown in Figure 1, nerol decreased contractile force of the isolated guinea pig atria (Figure 1(a), n = 5). This effect was dependent on nerol concentration (Figure 1(a), EC_50_ = 1.94 ± 0.2 mM, n = 5) and was almost completely reversible upon washout recovering up to 83.9 ± 4.1%.

Because the development of contractile force is known to rely on Ca^2+^ influx into the sarcoplasm from the extracellular milieu, we investigated whether nerol prevent Ca^2+^ activated force production. As depicted in Figure 1(b), the positive inotropic maximal response produced by increasing cumulatively extracellular Ca^2+^ was significantly diminished by nearly 25% by 3 mM nerol. Interestingly, EC_50_ which is a measure of responsiveness to Ca^2+^ was significantly altered by the presence of nerol (Figure 1(b), 2.2-fold increase). The EC_50_ of CaCl_2_ in control conditions was 1.7 ± 0.4 mM (Figure 1(b), closed squares, n = 8) and in the presence of nerol was 3.7 ± 0.5 mM (Figure 1(b), Open squares, n = 5, p<0.05). This enhancement of the EC_50_ of Ca^2+^ activation could be indicative of a reduction in Ca^2+^ entry.

We next considered whether nerol would inhibit L-type Ca^2+^ channels. Accordingly, L-type Ca^2+^ current (I_Ca,L_) was measured using whole-cell voltage-clamp technique in isolated guinea pig ventricular cardiomyocytes. As illustrated in Figure 1(c), 300 *μ*M nerol caused a significant decreased in peak I_Ca,L_. After 1.15 minutes of superfusion with nerol, a decrease of peak I_Ca,L_ from -4.0 ± 0.5 A/F to -1.6 ± 0.4 A/F was observed. The average decrease of peak I_Ca,L_ induced by nerol was 58.9 ± 6.3% (Figure 1(d), p<0.05, n = 7). These results indicate that Ca^2+^ influx was reduced by nerol through inhibition of L-type Ca^2+^ channels in guinea pig cardiomyocytes.

**Figure 2:**
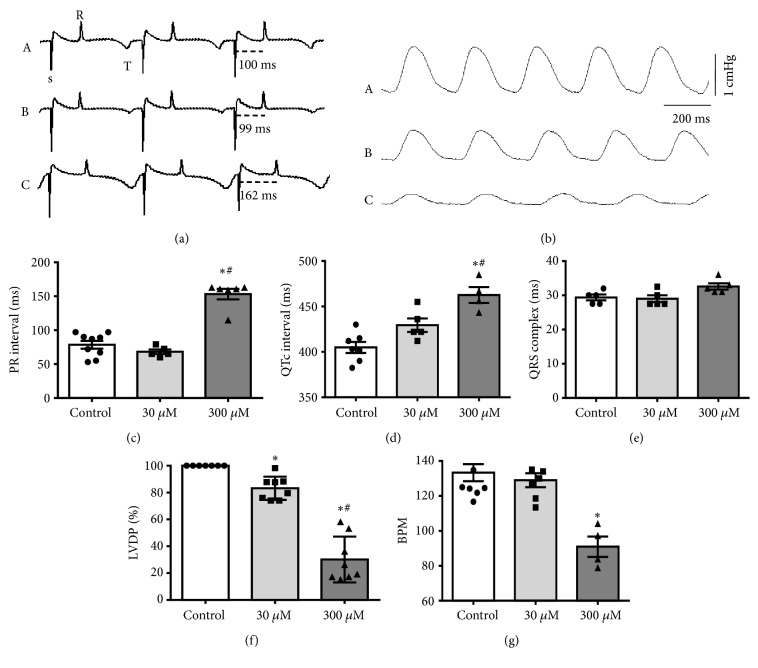
*Effect of nerol on the electrocardiographic parameters and left ventricular developed pressure (LVDP) in guinea pig isolated heart.* (a) Representative traces of ECG in control (A), 30 *μ*M (B), and 300 *μ*M nerol (C). (b) Representative traces of LVDP in control (A), after 10 min of perfusion with 30 *μ*M (B) and 300 *μ*M (C) nerol. (c) Effect of nerol on the PR interval, (d) QTc interval, (e) QRS complex duration, (f) LVDP, and (g) heart rate (BPM). Data are represented as means ± SEM (n = 4-9, *∗*p<0.05 versus control, #p<0.05 versus 30 *μ*M nerol). One-way ANOVA followed by Tukey's* post hoc* test.

We also investigated the effects of nerol in isolated heart recording simultaneously electrocardiogram (ECG, Figure 2(a)) and left ventricular developed pressure (LVDP, Figure 2(b)). ECG was recorded in electrically stimulated heart to permit measurement of ECG intervals and in spontaneously beating heart to evaluate heart rate. Figure 2(a) displays typical traces of ECG in control (Trace a) and during 30 *μ*M (Trace b) and 300 *μ*M nerol perfusion (Trace c). The results showed that 30 *μ*M nerol did not change PR interval (Figure 2(c)), QTc interval (Figure 2(d)), QRS complex duration (Figure 2(e)), and heart rate (Figure 2(g)). Important to note is the fact that at this concentration nerol decreased LVDP by only 17% (Figure 2(f)).

On the other hand, when using higher concentration, nerol (300 *μ*M) evoked an increase of PR interval (from 78.5 ± 5.7 ms to 153.2 ± 7.7 ms, p<0.05, Figure 2(c)) and QTc interval (from 404.9 ± 6.0 ms to 462.6 ± 8.8 ms, p<0.05, Figure 2(d)) without altering QRS complex duration (Figure 2(e)). Furthermore, this concentration promoted reduction of LVDP to 30.1 ± 6.0% (Figure 2(f)) and heart rate (from 133.4 ± 4.9 bpm to 90.9 ± 5.8 bpm, p<0.05, Figure 2(g)).

**Figure 3:**
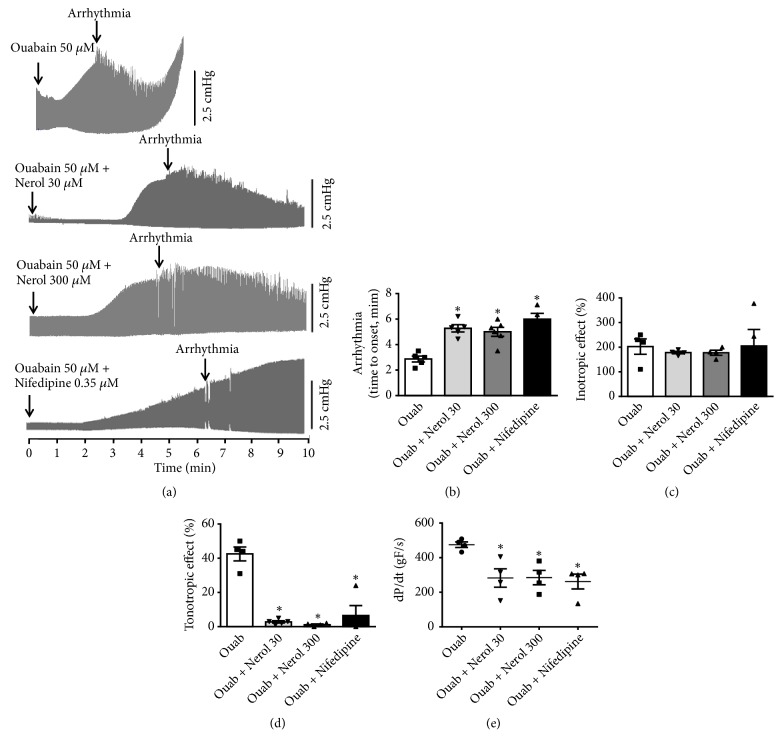
*Protective effects of nerol on ouabain-induced arrhythmias in guinea pig hearts.* (a) Representative recording of effects of ouabain (Ouab, 50 *μ*M) on left ventricular developed pressure (LVDP, Top panel), nerol (30 and 300 *μ*M) + ouabain (middle), and nifedipine (0.35 *μ*M) + ouabain (botton). Summary of effects of nerol or nifedipine preincubation on the onset time of arrhythmia (b), inotropic response (c), tonotropic effect (d), and inotropic response rate (dP/dt) induced by ouabain (e), respectively. Data are represented as means ± SEM (n = 5 - 6, *∗*p<0.05 versus Ouab). One-way ANOVA followed was by Tukey's* post hoc* test.

As L-type calcium channel blocker drugs are used as cardiac antiarrhythmics we reasoned that nerol could be of interest to study possible antiarrhythmic properties. The protective effects of nerol on ouabain-induced arrhythmias were examined in guinea pig hearts.  Figure 3(a) shows representative recording of ouabain (50 *μ*M) effects on LVDP (Top panel). As one would expect ouabain elicited a progressive increase in ventricular inotropic response. The systolic contractions became irregular (arrhythmic events) after 3.04 ± 0.25 min (onset of arrhythmia, n = 6). After this period, contractile force amplitude significantly decreased; arrhythmia then became severe and associated with strong diastolic contracture (tonotropic effect). Finally, cardiac arrest occurred at the end of 5 min. However, when ouabain (50 *μ*M) was added 15 min after the perfusion of 30 or 300 *μ*M nerol, the onset time of arrhythmia considerably delayed to 5.3 ± 0.28 min (p<0.05, n = 6) and 5.00 ± 0.35 min (p<0.05, n = 6) (Figure 3(a), middle panel), respectively. We took advantage of a well-known L-type Ca^2+^ channel inhibitor, nifedipine, used at 0.35 *μ*M to match a similar L-type Ca^2+^ current blockade as observed with nerol at 300 *μ*M. Therefore, this concentration of nifedipine would cause about 60% reduction of the L-type Ca^2+^ current [22]. This maneuver was done in order to evaluate the involvement of similar reduction of L-type Ca^2+^ current on antiarrhythmic effect (Figure 3(a), bottom panel). Importantly, nifedipine promoted a delay of 5.90 ± 0.50 min (n = 4) on the onset time of ouabain-triggered arrhythmia which is strikingly similar to that provoked by nerol. Figures 3(b), 3(c), 3(d), and 3(e) summarize the effects of nerol or nifedipine preincubation on the onset time of arrhythmia, inotropic response, tonotropic effect, and inotropic response rate (dP/dt) induced by ouabain, respectively. We conclude that nerol can mimic the nifedipine in abolition of responses induced by ouabain.

**Figure 4:**
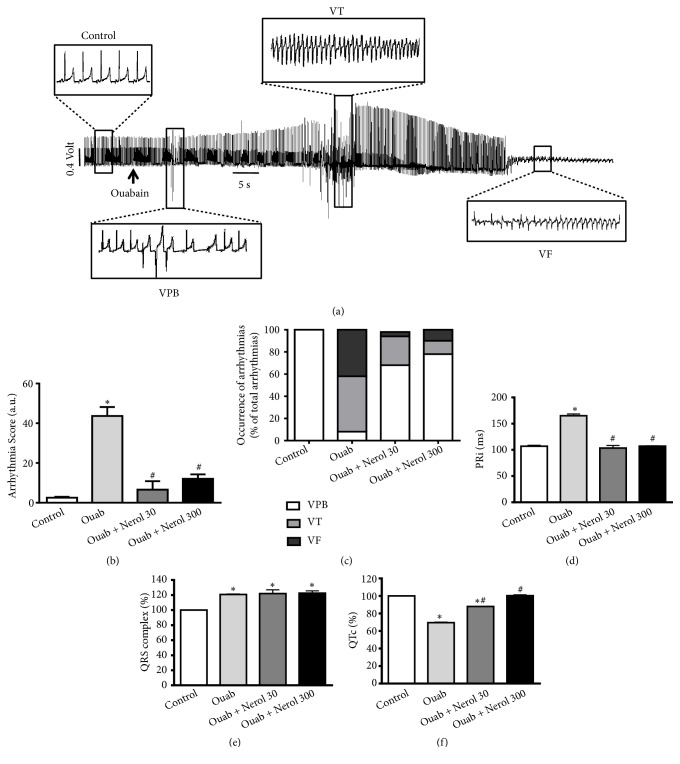
*Nerol attenuates cardiac arrhythmias and electrocardiographic (ECG) alterations induced by ouabain.* (a) Representative ECG recordings with 50 *μ*M ouabain (Ouab) evoking the occurrence of ventricular premature beatings (VPB), ventricular tachycardia (VT), and ventricular fibrillation (VF). (b) Arrhythmias score and (c) occurrence of arrhythmias (VPB, VT, VF). ECG parameters: PR interval (d), QRS complex (e), and QTc interval (f). Data are represented as means ± SEM (n = 4-7, *∗*p<0.05 versus control, # p<0.05 versus ouabain). One-way ANOVA followed by Tukey's* post hoc* test. Chi-squared test (c).

The data above are consistent with the idea of proposing nerol as an antiarrhythmogenic agent. The remaining experiments aim to clarify this possibility further. By analyzing electrocardiographic tracings, it was possible to classify the major types of arrhythmic events induced by ouabain. As demonstrated in Figure 4(a), 50 *μ*M ouabain evoked the occurrence of ventricular premature beatings (VPB), ventricular tachycardia (VT), and ventricular fibrillation (VF). The severity score of arrhythmias increased from 2.5 ± 0.6 a.u (control) to 43.6 ± 4.5 a.u after exposure to ouabain (Figure 4(b), *∗*p < 0.05). Notably, preincubation with 30 and 300 *μ*M nerol decreased significantly the severity score of arrhythmia to 6.6 ± 1.8 a.u and 12.2 ± 3.8 a.u, respectively (Figure 4(b), p < 0.05).  Figure 4(c) shows that in control conditions the VPB is by far the prevalent (100%) form of arrhythmia whereas when hearts were perfused with ouabain more severe types of arrhythmias were observed such as VT (50% occurrence) and VF (42% occurrence). Importantly, preincubation with nerol (30 and 300 *μ*M) attenuated the development of more severe arrhythmias as VT and VF being the ventricular arrhythmia; VPB was more prevalent. Ouabain, by itself, provoked an increase of PRi (54%, Figure 4(d)) and QRS complex duration (21%, Figure 4(e)) associated with reduction of QTc (30%, Figure 4(f)). Noticing that those ECG alterations are reported to be typical of digitalis toxicity [23, 24]. To our surprise, the increase of PRi and QTi and alterations previously described in ECG parameters were prevented by preincubation with 30 and 300 *μ*M nerol.

## 4. Discussion

The development of cardiac arrhythmias result from a number of different causes from genetic mutations to acquired cardiac diseases. Unequivocally they constitute a major cause of sudden death in the world [25, 26]. In the past few decades, it has become clear that cardiac arrhythmogenesis is related to ion channel dysfunctions and uncontrolled intracellular Ca^2+^ dynamics. It is, therefore, imperative to motivate studies looking for pharmacological agents that present cardioprotective effects against cardiac arrhythmias [5, 27, 28].

In this study, we used a combination of different approaches to investigate the mechanisms by which nerol acts controlling Ca^2+^ influx and the subsequent impact on ouabain-triggered arrhythmias. Our major findings are as follows: (a) nerol in lower concentrations (30 *μ*M) promotes small reduction of contractile response without alter ECG parameters and pacemaker activity; (b) nerol at 300 *μ*M inhibits L-type Ca^2+^ current by 60% and affect ECG, heart rate, and left ventricular performance; (c) nerol, in both concentrations investigated, suppressed ouabain-triggered arrhythmias; and (d) nerol protects the heart against ventricular tachycardia and ventricular fibrillation. These findings, altogether, are consistent with the idea of proposing nerol as an antiarrhythmogenic agent.

To our surprise, there are fewer studies using nerol (trans isomer) than geraniol (cis isomer). Nonetheless, there are numerous reports on the antitumoral [29], antioxidant [30], and anti-inflammatory properties of these monoterpenes. Specifically in the case of nerol limited information is available on its effect on cardiac myocytes [31].

In mammalian heart, several routes can lead to reduction of cardiac contractility and as it is well known, intracellular Ca^2+^ is central to regulate contractile force in the heart. In fact, our results show that nerol decreases Ca^2+^ entry into cardiomyocytes through inhibition of L-type Ca^2+^ channels reducing contractile force. Important to note at this point that nerol at 300 *μ*M mediates 70% of reduction on contractile force and 60% blockade of L-type Ca^2+^ current. This allowed us to study further to better characterize the pharmacological effects of nerol. Furthermore, in this concentration, a decrease in pacemaker activity (31%) associated with increase of both PRi and QTi at baseline conditions was observed. These results were somehow expected because previously Menezes-Filho et al. [9] reported that geraniol (a nerol geometric isomer) elicited PR and QT interval increase, reduced pacemaker activity (~16%), and markedly reduced LVDP (~83%). A novel finding in this study is that nerol, in lower concentration, did not affect pacemaker activity or ECG major parameters but promoted a small reduction on LVDP (17%).

Stability of intracellular Ca^2+^ dynamics is intimately connected to I_Ca,L_, Na^+^/Ca^2+^ exchange (NCX), Sarco(Endo)plasmic Reticulum Ca^2+^-ATPase (SERCA2a), and RyR2 in cardiomyocytes, being the key regulators of cardiac contractility. However, any source of instability can provoke arrhythmias [32]. Arrhythmia as induced by ouabain is originated by several mechanisms. The most accepted starts with Na^+^/K^+^ pump blockade by cardiac glycosides (such as ouabain) which increases [Na^+^]i, which enhances cell contractility by increasing intracellular [Ca^2+^] due to NCX [33]. This may be explained by reduced Ca^2+^ efflux by the NCX as [Na^+^]i rises. In other words, less Ca^2+^ efflux for a given Ca^2+^ influx would increase intracellular Ca^2+^. The increased [Na^+^]i could also increase Ca^2+^ influx via reverse-mode of NCX [34, 35] producing Ca^2+^ overload. During Ca^2+^ overload oscillatory Ca^2+^ releases from the SR lead to membrane depolarization favoring triggered arrhythmias. To gain more insight into nerol antiarrhythmogenic potential we made use of the ouabain-triggered arrhythmia model in guinea pig.

Our results indicate that tonotropic effect caused by ouabain is completely abolished by nerol or nifedipine suggesting that ouabain-elicited tonotropic effect does depend on Ca^2+^ influx through L-type Ca^2+^ channels. We can conclude that nerol interacts with and inhibits L-type Ca^2+^ channels. Nerol and nifedipine slowed down LVDP rate (dP/dt) arguing in favor of the notion that L-type Ca^2+^ current is participating on early phase of inotropic response.

Several groups have previously demonstrated that the positive inotropism observed during the exposure to ouabain is mostly related to Ca^2+^ influx through NCX when intracellular [Na^+^]i increases as a result of Na^+^/K^+^ ATPase inhibition. Nerol at 30 and 300 *μ*M delayed the onset time for cardiac arrhythmias but the ouabain-triggered inotropy remained largely intact (see Figure 3(b)). These results suggest that nerol does not affect, directly or indirectly, NCX mediated Ca^2+^ dynamics. Furthermore, nerol decreased the occurrence of more severe arrhythmias such as tachycardia and ventricular fibrillation.

In our study, nerol corrected key adverse effects of ouabain-triggered arrhythmias: it abolished heart diastolic contracture, it recovered electrical activity, and it reduced the propensity to generate arrhythmias. This is the first study to actually demonstrate that, mechanistically, nerol at low concentration can preserve inotropy and the electrical properties of cardiac muscle while at the same time acting as antiarrhythmic agent. Taken all together, nerol may be useful adjunct to digitalis treatment and justifies further investigation.

## 5. Limitations

Small animal models are commonly used in cardiovascular research showing advantages and disadvantages [36]. The present study used guinea pig as a model because its isolated cardiomyocytes' action potential waveform and calcium handling resembles that encountered in human ventricular cardiomyocytes. In accordance with this statement, the effect of ouabain, used to induce arrhythmias, is more pronounced in guinea pigs because of higher relative contribution of NCX (28%) in removing intracellular calcium during diastole when compared to rats (7%) [37]. Although the animal model has similarities, we must be cautious in translating the results to humans. As nerol presented antiarrhythmic effects in a very specific model (ouabain-triggered arrhythmias) we intend to continue this investigation using other models to induce cardiac arrhythmias that could be closer to that of humans and therefore establish a more general mechanism to impair arrhythmogenesis.

## Data Availability

The data used to support the findings of this study are available from the corresponding author upon request.
